# Increased expression of NLRP3 associated with elevated levels of HMGB1 in children with febrile seizures: a case–control study

**DOI:** 10.1186/s12887-024-04533-4

**Published:** 2024-01-13

**Authors:** Xing-Guang Ye, Feng-Zhi She, Dong-Ni Yu, Li-Qian Wu, Yan Tang, Ben-Ze Wu, Shi-Wei Dong, Jie-Min Dai, Xing Zhou, Zhi-Gang Liu

**Affiliations:** 1 Department of Pediatrics, Foshan Women and Children Hospital, Foshan, 528000 Guangdong China; 2 The Second School of Clinical Medicine, Southern Medical University, Guangzhou, China

**Keywords:** HMGB1, NLRP3, IL-1β, Inflammatory cytokines, Febrile seizures

## Abstract

**Background:**

High mobility group box-1 (HMGB1) is an endogenous danger signal that mediates activation of the innate immune response including NLR pyrin domain containing 3 (NLRP3) inflammasome activation and proinflammatory cytokine release. Although HMGB1 and NLRP3 have been implicated in the pathophysiology of seizures, the correlation between HMGB1 and NLRP3 expression has not been determined in children with febrile seizures (FS). To explore the relationship between extra-cellular HMGB1 and NLRP3 in children with FS, we analyzed serum HMGB1, NLRP3, caspase-1, and proinflammatory cytokines in patients with FS.

**Methods:**

Thirty children with FS and thirty age-matched febrile controls were included in this study. Blood was obtained from the children with FS within 1 h of the time of the seizure; subsequently, the serum contents of HMGB1, NLRP3, caspase-1, interleukin (IL)-1β, interleukin (IL)-6, and tumour necrosis factor-α (TNF-α) were determined by enzyme-linked immunosorbent assay. The Mann‒Whitney *U* test was used to compare serum cytokine levels between FS patients and controls. Spearman’s rank correlation coefficient was calculated to detect significant correlations between cytokine levels.

**Results:**

Serum levels of HMGB1, NLRP3, caspase-1, IL-1β, IL-6, and TNF-α were significantly higher in FS patients than in febrile controls (*p* < 0.05). Serum levels of HMGB1 were significantly correlated with levels of NLRP3 and caspase-1 (both, *p* < 0.05). Serum levels of caspase-1 were significantly correlated with levels of IL-1β (*p* < 0.05). Serum levels of IL-1β were significantly correlated with levels of IL-6 and TNF-α (*p* < 0.05).

**Conclusions:**

HMGB1 is up-regulated in the peripheral serum of FS patients, which may be responsible, at least in part, for the increased expression of NLRP3 and Caspase-1. Increased expression of caspase-1 was significantly associated with elevated serum levels of IL-1β. Given that activated Caspase-1 directly regulates the expression of mature IL-1β and positively correlates with activation of the NLRP3 inflammasome, our data suggest that increased levels of peripheral HMGB1 possibly mediate IL-1β secretion through the activation of the NLRP3 inflammasome in children with FS. Thus, both HMGB1 and NLRP3 might be potential targets for preventing or limiting FS.

## Background

Febrile seizures (FS) are the most common type of convulsions in infants and children and typically occur in association with a fever more than 100.4°F (38 °C) in children 6 months to 5 years of age, who have no evidence of any central nervous system infection or metabolic disturbance. Its overall prevalence in children is approximately 2%-14% worldwide [1]. Although single short FS (generalized seizures lasting < 15 min) are generally benign, prolonged FS (pFS) (FS lasting > 15 min) are more likely to develop into temporal lobe epilepsy (TLE) later in life [2–6]. Retrospective studies have shown that 30%-60% of patients with TLE have a history of pFS [7]. Therefore, understanding the pathogenesis of FS is clinically important, because, if it is associated with subsequent epilepsy, then predictive biomarkers and preventive therapies might be feasible.

High mobility group box 1 (HMGB1) is a highly conserved, ubiquitously expressed nonhistone DNA-binding protein present in eukaryotic cells that functions in stabilizing nucleosomes and regulating gene transcription [8]. Previous studies have revealed increased expression levels of serum HMGB1 in FS patients [9–11]. Ito et al. found that HMGB1 enhances hyperthermia-induced seizures, contributes to FS pathogenesis and plays an important role in the acquired epileptogenesis of secondary epilepsy associated with pFS [12], indicating that HMGB1 related signalling contributes to the generation of FS in children. Furthermore, Choi and colleagues found that increased expression of HMGB1 was associated with elevated serum levels of interleukin (IL)-1β in children who had FS [11]. Yang and colleagues found that increased expression levels of HMGB1 and toll-like receptor (TLR)-4 showed a positive correlation with elevated serum levels of tumour necrosis factor-α (TNF-α) and IL-1β in a rat model and in children with TLE [13]. Taken together, the above data indicate a correlation between HMGB1 expression and IL-1β production. However, the nature of the link between HMGB1 and IL-1β has not been clarified in children with FS.

The role of HMGB1 and IL-1β in generating and perpetuating seizures is well-documented [14]. Physiologically, HMGB1 resides in the nucleus translocates to the cytosol under conditions of stress and is subsequently released into the extracellular space [15]. Once released into the extracellular space, HMGB1 protein serves as a typical alarmin or damage-associated molecular pattern (DAMP) that binds to cell membrane pattern recognition receptors (PRRs), including TLR2, TLR4 and the receptor for advanced glycation end products (RAGE), which are predominantly expressed by activated monocytes, macrophages, T-lymphocytes in plasma, microglia in the central nervous system [16]. Activation of TLR2 and TLR4 causes the recruitment of MyD88 to activate several mitogen-activated protein kinases (MAPKs) that activate the downstream transcription factor nuclear factor kappa B (NF-κB). Activated NF-κB moves into the nucleus and promotes the formation of the NOD-like receptor family pyrin domain containing 3 (NLRP3) inflammasome, thus enhancing the release of the proinflammatory cytokine IL-1β [17–19]. Therefore, it was speculated that the extracellular HMGB1 activated NLRP3 inflammasome possibly mediates IL-1β secretion in children with FS.

Given the correlation between HMGB1 and the NLRP3 inflammasome, the aim of the current study was to investigate whether HMGB1-induced activation of the NLRP3 inflammasome contributes to generation of FS by evaluating the protein expression levels of HMGB1, NLRP3, caspase-1, IL-1β, IL-6, and TNF-α in the peripheral serum of FS patients.

## Methods

### Participants

A total of 30 FS patients (aged 6 months to 5 years) who visited the Department of Paediatrics or or Emergency Department of Foshan Women and Children Hospital from January 2019 and April 2020 were included in this study (Table 1). All individuals enrolled were unrelated ethnic Han Chinese who lived in southern China. None of the biological grandparents of the participants were from other ethnicities. Peripheral blood was obtained from patients within 1 h of the time of seizure, and serum was immediately separated and frozen for subsequent cytokine assays. Patient inclusion criteria were age between 6 months and 5 years, body temperature ≥ 38.5 °C, and patients with conditions known or suspected to cause seizures without fever were systematically excluded as in our previous report [20].

**Table 1:** Clinical findings of febrile seizures and control children

Variables	Febrile seizures (*N* = 30)	Febrile controls (*N* = 30)	*P* Value
Male/Female	20/10	16/14	0.292
Age (months)^a^	22.67 ± 11.08	28.33 ± 16.85	0.129
Severity of temperature (℃)^a^	39.16 ± 0.50	38.95 ± 0.61	0.216
C-reactive protein (mg/l)^a^	5.50 ± 8.85	6.57 ± 10.58	0.673
Leukocytes (× 10^9^/l)	10.65 ± 4.34	10.53 ± 6.57	0.936
Etiology of infection (viral/bacterial)	21/9	24/6	0.371
Duration of seizure			
< 5 min	22		
5-15 min	8		
> 15 min	0		
Number of seizure			
1	22		
2	6		
3	2		

^a^Mean ± Standard deviation

Clinical data for familial FS history, previous FS attacks, and the duration and semiology of FS were obtained from the patients’ parents. Family history was regarded as positive when FS occurred in first-degree relatives. Laboratory findings, including complete blood counts, blood chemistry, and C-reactive protein levels, were checked at the time of seizure. Control samples were collected from children with febrile illness, without convulsion. Control groups were matched for age and temperature criteria and had no convulsions during febrile illness and no known history of previous FS. Thirty controls were included in the final analysis. Control blood serum was collected and frozen as described above. A diagnosis of FS was determined according to the International Classification of Diseases; Ninth Revision (ICD-9) codes (ICD-9 780.31, 780.32). All patients were followed for more than 1 year.

The study was approved by the Ethics Committee of Foshan Women and Children Hospital (Approved number: FSFY-MEC-2018–016). All experiments and methods were performed in accordance with the relevant guidelines and regulations. Informed consent was obtained from the patients’ legal guardians.

### Cytokine measurement

Four millilitres of blood were taken from the peripheral vessels of children in all the groups, and serum was obtained by centrifugation at 4000 rpm for 5 min at 4 °C. The serum was then added to acid-washed tubes and stored in a refrigerator at -80 °C until assay. Serum levels of HMGB1, NLRP3, caspase-1, and proinflammatory cytokines, including, IL-1β, IL-6, and TNF-α, were examined in FS patients and febrile controls using commercial enzyme-linked immunosorbent assay kits according to the manufacturer’s instructions (Cusabio Biotech, Wuhan, China).

### Statistical analysis

Statistical analyses were performed using SPSS Statistics 19.0 for Windows (SPSS Inc., Chicago, IL, USA). The chi-square test or t test was used for the comparison of clinical characteristics between FS patients and the controls. The Mann‒Whitney *U* test was used to compare serum cytokine levels and laboratory findings between FS patients and controls. Spearman’s rank correlation coefficient was calculated to detect significant correlations between cytokine levels. GraphPad Prism v.7.0 (GraphPad Software Inc., San Diego, CA, USA) was used to perform the above tests. Values are expressed as means. Statistical significance was defined as *P* < 0.05.

## Results

Table 1 shows the comparison of the selected patients’ clinical data. Thirty children with FS and 30 age matched control children with febrile illness without convulsion were included in this study. The mean age was 22.67 ± 11.08 months in the FS group and 28.33 ± 16.85 months in the febrile control group. FS was more prevalent in boys than in girls (66.7% vs. 33.3%, respectively). All patients had their first FS attack and 73.3% (22/30) of patients had a duration of seizure < 5 min and a single seizure. There were no statistically significant differences between the two groups with respect to sex, age, severity of temperature, C-reactive protein levels, leukocytes or type of febrile disease (*p* > 0.05).

**Fig. 1:**
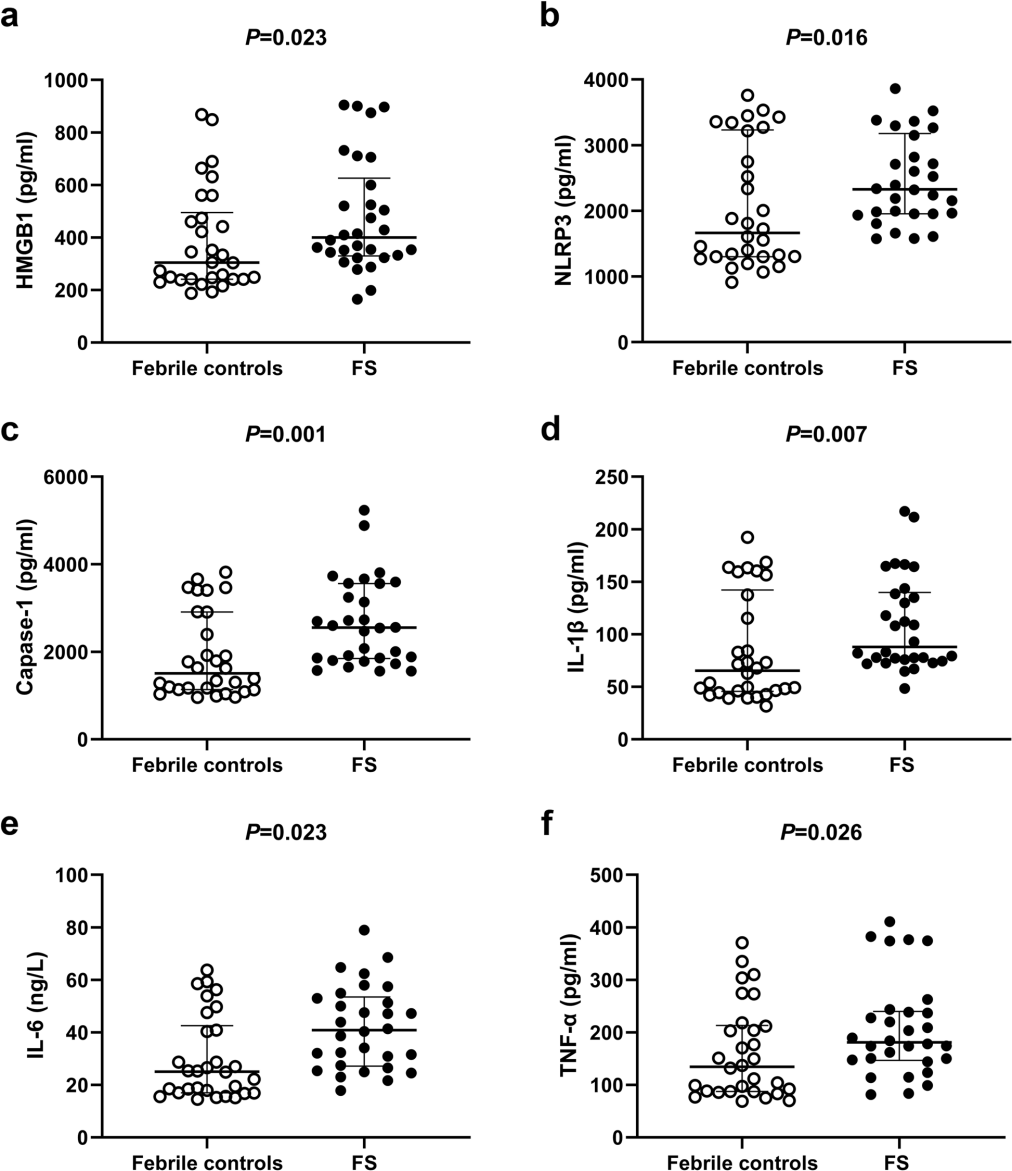
Comparison of serum levels of HMGB1 (**a**), NLRP3 (**b**), caspase-1 (**c**), IL-1β (**d**), IL-6 (**e**), and TNF-α (**f**) between the febrile seizure group and the control group. The median (interquartile range) values are indicated by three parallel lines. Analysis of serum cytokine levels between the two groups was performed by the Mann‒Whitney U test. HMGB1, NLRP3, caspase-1, IL-1β, IL-6, and TNF-α levels were significantly higher in the febrile seizure group than in the control group (*p* < 0.05 indicates a significant difference)

**Fig. 2:**
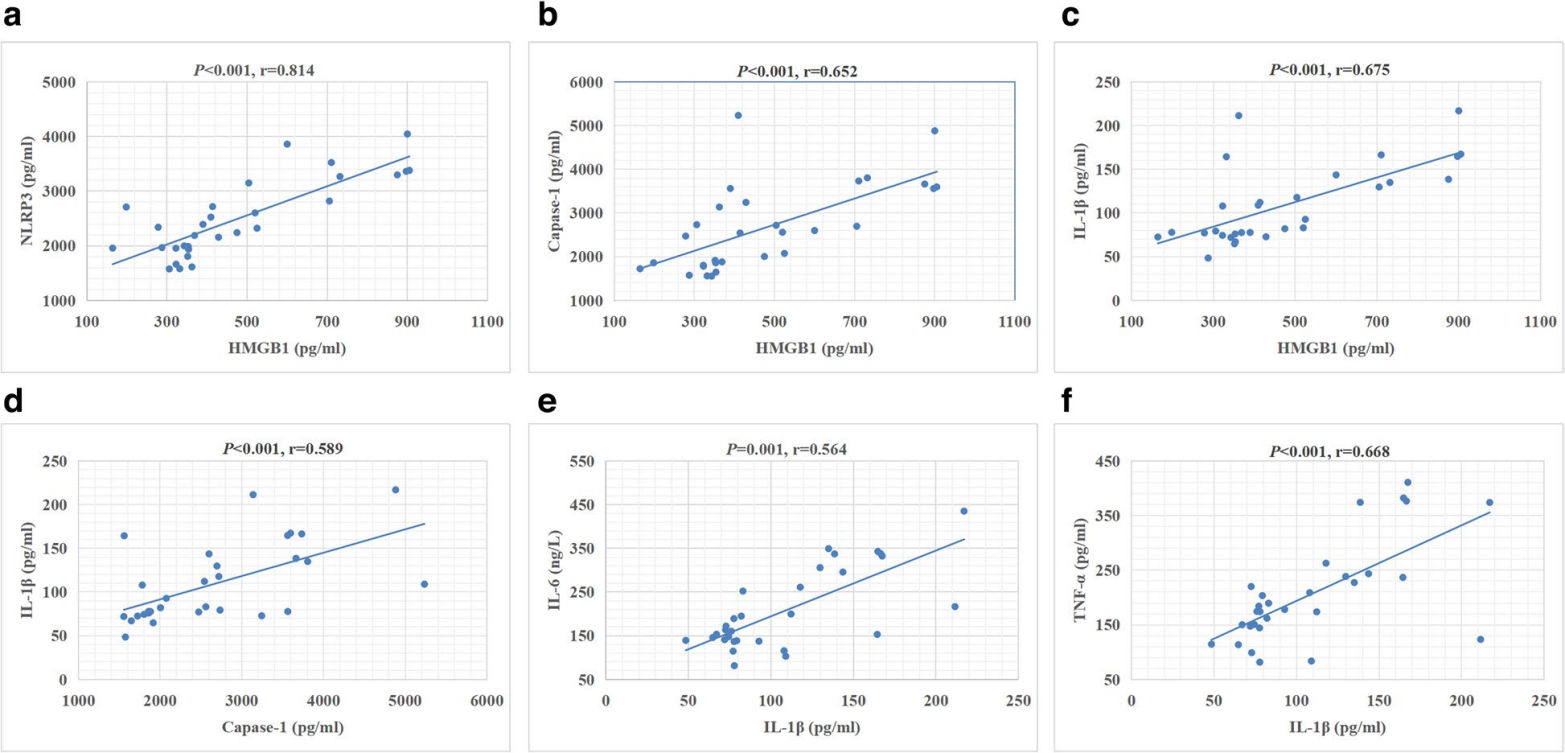
Correlation between serum cytokine levels in the febrile seizure group. (**a-f**) Correlation between serum levels of NLRP3 and HMGB1 (**a**), caspase-1 and HMGB1 (**b**), IL-1β and HMGB1 (**c**), IL-1β and caspase-1 (**d**), IL-6 and IL-1β (**e**), and TNF-α and IL-1β (**f**) in children with febrile seizure. HMGB1 levels were significantly correlated with NLRP3, caspase-1, and IL-1β levels (all, *p* < 0.05, r = 0.814, *r* = 0.652, and *r* = 0.675, respectively). Caspase-1 levels were significantly correlated with IL-1β levels (*p* < 0.05, *r* = 0.589). IL-1β levels were significantly correlated with IL-6 and TNF-α levels (both, *p* < 0.05, *r* = 0.564 and 0.668, respectively)

When we compared the FS group with the febrile control group, the serum levels of HMGB1 (Fig. 1a, *p* = 0.023), NLRP3 (Fig. 1b, *p* = 0.016), caspase-1 (Fig. 1c, *p* = 0.001), IL-1β (Fig. 1d, *p* = 0.007), IL-6 (Fig. 1e, *p* = 0.023), and TNF-α (Fig. 1f, *p* = 0.026) were significantly higher in the FS group than in the febrile control group (Table 2). Additionally, HMGB1 serum levels were significantly correlated with NLRP3, caspase-1, and IL-1β (Fig. 2a, b, and c; *r* = 0.814, *r *= 0.652, and *r* = 0.675, respectively, all *p* < 0.001). Caspase-1 serum levels were significantly correlated with IL-1β expression (Fig. 2D, *r* = 0.589; *p* < 0.001). Serum IL-1β levels were significantly correlated with IL-6 and TNF-α levels (Fig. 2E and F; *r* = 0.564 and *r* = 0.668, respectively, both *p* < 0.001).

**Table 2:** Comparison of HMGB1, NLRP3, Capase-1, and cytokine levels between the febrile seizures group and febrile control group

Variables	FS group^a^ (*N* = 30)	Control group^a^ (*N* = 30)	*P-*Value
HMGB1 (pg/ml)	399.84 (329.69–626.52)	304.56 (240.86–495.16)	0.023*
NLRP3 (pg/ml)	2330.15 (1956.64–3179.77)	1666.14 (1302.69–3231.45)	0.016*
Capase-1 (pg/ml)	2550.69 (1845.07–3560.79)	1504.81 (1134.57–2909.78)	0.001*
IL-1β (pg/ml)	87.90 (75.58–139.83)	65.31 (45.66–142.17)	0.007*
IL-6 (pg/ml)	40.87 (27.15–53.46)	25.06 (16.94–42.56)	0.003*
TNF-α (pg/ml)	181.05 (146.58–239.76)	134.39 (87.23–213.34)	0.026*

*FS* febrile seizure, *HMGB1* high mobility group box-1, *IL-1β* interleukin-1beta, *N* number, *TNF-α* tumor necrosis factor α. The *P*-value is for Mann–Whitney U-test

^a^Median (interquartile range)

^*^Indicates a significant difference

In this study, all the cases with FS were divided into simple FS according to the recorded seizure data (generalized and non-recurrent seizure within 24 h, and duration of seizure ≤ 15 min). Then we divided all the cases with FS into two groups: group1 (duration of seizure ≤ 5 min) and group2 (duration of seizure > 5 min). There were no statistically significant differences between the groups with respect to level of HMGB1, NLRP3, Caspase-1, IL-1β, IL-6, and TNF-a (*p* > 0.05) (Table 3).

**Table 3:** Comparison of HMGB1, NLRP3, Capase-1, and cytokine levels in patients with different duration of seizure

Variables	duration of seizure ≤ 5 min (*N* = 22)^a^	duration of seizure > 5minnutes (*N* = 8)^a^	*P Value*
HMGB1 (pg/ml)	411.9 (338.0–626.5)	357.8 (325.1–656.2)	0.798
NLRP3 (pg/ml)	2365 (1964–3187)	2071 (1625–3128)	0.440
Capase-1 (pg/ml)	2551 (1862–3571)	2498 (1801–3481)	0.977
IL-1β (pg/ml)	82.55 (73.92–131.97)	121.5 (76.4–164.7)	0.238
IL-6 (pg/ml)	40.87 (26.90–55.57)	37.72 (27.53–52.55)	0.842
TNF-α (pg/ml)	181.1 (146.6–284.4)	191.6 (135.9–234.4)	0.842

*FS* febrile seizure, *HMGB1* high mobility group box-1, *IL-1β* interleukin-1beta, *N* number, *TNF-α* tumor necrosis factor α. The *P*-value is for Mann–Whitney U-test

^a^Median (interquartile range)

## Discussion

In the current study, we evaluated the expression of HMGB1 and the NLRP3 inflammasome alongside caspase-1 and IL-1β in FS patients compared with febrile controls. Despite their role in triggering the neuroinflammatory response, HMGB1 and the NLRP3 inflammasome have been poorly studied in FS. We confirmed the results of previous studies of increased HMGB1 and NLRP3 expression in FS [11, 20], reporting that increased expression of NLRP3 was associated with elevated plasma levels of HMGB1 in FS for the first time. Moreover, serum levels of other proinflammatory cytokines, including IL-1β, TNF-α, and IL-6 were significantly higher among patients with FS.

Over the past two decades, the neuroinflammatory response and the release of proinflammatory,cytokines including HMGB1, IL-1β, TNF-α, and IL-6, have been implicated in the pathophysiology of FS [11, 21–24]. Of these proinflammatory cytokines, HMGB1 and IL-1β are key initiators of neuroinflammation contributing not only to the generation of FS but also to epileptogenesis after prolonged FS [12, 13, 25–30]. Experimental studies have shown that increased levels of HMGB1 and IL-1β contribute to chronic inflammation, neuronal excitotoxicity and a reduction in the seizure threshold [12, 14, 17, 27–29, 31–33]. Moreover, HMGB1 and IL-1β levels are increased in epileptogenic brain tissue [13, 28, 30]. Interestingly, the levels of HMGB1 were positively correlated with the serum levels of IL-1β in a rat model and in children with TLE, while HMGB1 treatment of hippocampal neurons induced a significant increase in the levels of IL-1β [13]. These data suggested that HMGB1-IL-1β network may contribute to the generation of seizures. In this study, we showed that patients with FS also display higher circulating (i.e. plasma) levels of HMGB1 and IL-1β. We also found that increased expression of HMGB1 was associated with elevated serum levels of IL-1β in peripheral blood after FS in children, indicating that there is a correlation between HMGB1 and IL-1β in children with FS. However, it was unclear how HMGB1 induces IL-1β expression.

HMGB1 is a highly conserved, ubiquitously expressed protein that can serve as a representative DAMP [33]. DAMPs are pivotal for the activation of NLRP3 inflammasome pathways [34]. Under normal circumstances, microglia and astrocytes express insufficient amounts and the NLRP3 inflammasome exists in an inactive form. When cells are subjected to specific stimuli, such as lipopolysaccharide (LPS), the NLRP3 inflammasome can be activated [35]. Assembly and activation of the NLRP3 inflammasome requires two functionally distinct steps: ‘priming’ and ‘activation’ [36]. Recent studies have demonstrated that HMGB1 can stimulate increased expression of NLRP3 to a critical level necessary for inflammasome formation, thus causing the priming process of the NLRP3 inflammasome via the TLR4/NF-κB signaling pathway [37], and causing sustained activation of the NLRP3 inflammasome [32, 38]. NLRP3 inflammasome-dependent caspase-1 activation is an important pathway related to IL-1β release [39] and has been implicated in the pathophysiology of neurological diseases, including Parkinson’s disease, Alzheimer’s disease, multiple sclerosis, and epilepsy [40–42]. In this study, we demonstrated a significant increase in the expression of NLRP3 in peripheral blood after FS in children, and a significant correlation between caspase-1 expression and serum levels of IL-1β, as described in our previous study [20]. As expected, we also observed a positive correlation between HMGB1 and NLRP3 expression, and a positive correlation between HMGB1 and caspase-1. Given that activated caspase-1 directly regulates the expression of mature IL-1β and positively correlates with activation of the NLRP3 inflammasome [20], our results suggest that increased levels of peripheral HMGB1 possibly mediate IL-1β secretion through the activation of the NLRP3 inflammasome in children with FS, and HMGB1/NLRP3 inflammasome/caspase-1/IL-1β pathway may contribute to the generation of FS in children. Further studies are needed to verify the mechanism.

In addition to IL-1β and HMGB1, inflammatory cytokines, including IL-6 and TNF-α, might also have facilitatory effects on the development of FS [43]. IL-1β can bind to IL-1 receptor type 1 (IL-1R1), a Toll receptor family member, and induce the transcription of various genes that encode several downstream mediators of inflammation, including TNF-α and IL-6, via an NF-κB-related pathway [31, 44]. In this study, we found that IL-6 and TNF-α serum levels were significantly higher in FS patients than in febrile children without seizures, and IL-6 and TNF-α levels positively correlated with the serum levels of IL-1β in children with FS. These observations, together with experimental animal studies in which transgenic mice overexpressing high amounts of IL-6 or TNF-α in astrocytes were reported to have increased seizure susceptibility [45–48], support the possibility that IL-1β is a pluripotent proinflammatory cytokine and the key interleukin involved in FS pathogenesis.

The current study has several limitations. First, the limited number of samples weakens the strength of this study. Second, levels of the HMGB1, NLRP3, Caspase-1 and the proinflammatory cytokines were not measured in the cerebrospinal fluid, which would make a significant contribution to the evaluation. Last, the follow-up time was relatively short, no valuable data from this follow-up were obtained. Long-term follow-up data could provide valuable insights into the prognosis and outcomes of children with FS.

## Conclusions

In conclusion, our present study showed that HMGB1 is up-regulated in peripheral serum of FS patients, which may be responsible, at least in part, for the increased expression of NLRP3 and caspase-1. Increased expression of caspase-1 was significantly associated with elevated serum levels of IL-1β. Our data suggest that increased levels of peripheral HMGB1 possibly mediate IL-1β secretion through the activation of the NLRP3 inflammasome in peripheral blood after FS. Thus, both HMGB1 and the NLRP3 inflammasome might be potential targets for preventing or limiting FS.

## Data Availability

The datasets for this article are not publicly available due to concerns regarding participant/patient anonymity. Requests to access the datasets should be directed to the corresponding author.

## Abbreviations

DAMPs: Damage associated molecular patterns
FS: Febrile seizures
HMGB1: High mobility group box-1
IL-1β: Interleukin-1β
IL-6: Interleukin-6
LPS: Lipopolysaccharide
MAPKs: Mitogen-activated protein kinases
NLRP3: NLR pyrin domain containing 3
NF-κB: Nuclear factor kappa B
PRRs: Pattern recognition receptors
RAGE: Receptor for advanced glycation end products
TNF-α: Tumor necrosis factor-α
TLE: Temporal lobe epilepsy
TLR: Toll-like receptor
